# Specific detection of H5N1 avian influenza A virus in field specimens by a one-step RT-PCR assay

**DOI:** 10.1186/1471-2334-6-40

**Published:** 2006-03-02

**Authors:** Lisa FP Ng, Ian Barr, Tung Nguyen, Suriani Mohd Noor, Rosemary Sok-Pin Tan, Lora V Agathe, Sanjay Gupta, Hassuzana Khalil, Thanh Long To, Sharifah Syed Hassan, Ee-Chee Ren

**Affiliations:** 1 Genome Institute of Singapore, 60 Biopolis Street, Genome, #02-01, 138672, Singapore; 2 WHO Influenza Centre, 45 Poplar Road, Parkville, Melbourne, 3052, Australia; 3 Virology Section, National Centre for Veterinary Diagnostics, Department of Animal Health, Vietnam; 4 Virology Section, Veterinary Research Institute, 31400, Ipoh, Perak, Malaysia; 5 Veredus Laboratories Pte Ltd, 83 Science Park Drive, #03-02A The Curie, Singapore Science Park 1, 118259, Singapore; 6 Department of Microbiology, Faculty of Medicine, National University of Singapore, Block MD4, 5 Science Drive 2, 117597, Singapore

## Abstract

**Background:**

Continuous outbreaks of the highly pathogenic H5N1 avian influenza A in Asia has resulted in an urgent effort to improve current diagnostics to aid containment of the virus and lower the threat of a influenza pandemic. We report here the development of a PCR-based assay that is highly specific for the H5N1 avian influenza A virus.

**Methods:**

A one-step reverse-transcription PCR assay was developed to detect the H5N1 avian influenza A virus. The specificity of the assay was shown by testing sub-types of influenza A virus and other viral and bacterial pathogens; and on field samples.

**Results:**

Validation on 145 field specimens from Vietnam and Malaysia showed that the assay was specific without cross reactivity to a number of other infuenza strains as well as human respiratory related pathogens. Detection was 100% from allantoic fluid in H5N1 positive samples, suggesting it to be a reliable sampling source for accurate detection.

**Conclusion:**

The assay developed from this study indicates that the primers are specific for the H5N1 influenza virus. As shown by the field tested results, this assay would be highly useful as a diagnostic tool to help identify and control influenza epidemics.

## Background

Influenza A virus infects many animals such as humans, pigs, horses, marine mammals, and birds [1]. In avian species, most influenza virus infections cause mild localized infections of the respiratory and intestinal tract, but highly pathogenic strains such as H5N1 cause system infections in which mortality may reach 100% [2]. In humans, influenza viruses cause a highly contagious acute respiratory disease that resulted in epidemic and pandemic disease in humans [3].

Three types of influenza viruses, types A, B, and C are known and they belong to a family of single-stranded negative-sense enveloped RNA viruses called *Orthomyxoviridae *[4]. The viral genome is comprised of eight RNA segments (seven in Type C). Influenza A viruses can be classified into subtypes based on antigenic differences in the two surface glycoproteins, namely, hemagglutinin (HA) and neuraminidase (NA) which are required for viral attachment and cellular release. Other major viral proteins include the nucleoprotein (NP) which is the main structural protein, membrane proteins (M1 and M2), polymerase proteins (PA, PB1 and PB2), and non-structural proteins (NS1 and NS2). Currently, sixteen subtypes of HA (H1-H16) and nine NA (N1-N9) antigenic variants are known in influenza A virus mostly related with veterinary significance, with only three subtypes circulating in humans (H1N1, H1N2, and H3N2). However, in recent years, the pathogenic H5N1 subtype of avian influenza A has been reported to cross the species barrier and infect humans as documented in Hong Kong in 1997 and 2003 [5-7]. Since late 2003, the H5N1 avian A influenza in poultry reached epidemic proportions with reports of serious outbreaks in several Asian countries including Vietnam, Thailand, South Korea, Laos, Cambodia, Indonesia, Japan and Malaysia [8,9] that resulted in massive culling of millions of poultry which had severe economic repercussions.

As a result, H5N1 avian influenza A virus represents a potential danger to human health not only in Asia but to the world. Therefore, in addition to containment procedures, sensitive detection assays for early diagnosis are vital to lower the chances of spread and reduce the risk of development into an epidemic. Current methods employed to detect H5N1 subtypes include various polymerase chain reaction (PCR) assays [10-12,7] and antigen tests using various fluorescence and enzyme-linked immunoassays [9]. However, these assays are reported to be low in specificity and sensitivity, and clinically, the low sensitivity of these diagnostics may limit the usefulness for reliable detection of influenza A (H5N1) virus in humans [9]. Therefore, there is an urgent need for improved, validated, sensitive diagnostic tests for rapid and early diagnosis. In this study, we describe the development of a nucleic acid detection test that is rapid, specific and sensitive, thus allowing greatly improved detection of the H5N1 avian influenza A virus.

## Methods

### RNA isolation

Extraction of total RNA was performed following manufacturers' protocol from QIAamp Viral RNA Mini Kit (Qiagen, Germany) and TRIZOL (Invitrogen, USA) using all necessary safety precautions. The resultant RNA was dissolved in 20 μl of RNase-free water.

### PCR

2 μl of RNA was used in 25 μl reaction mixtures using the One-Step reverse transcription (RT)-PCR system (Qiagen, Germany) with H5N1 specific primers (forward primer: 5'-ACTATGAAGAATTGAAACACCT-3' and reverse primer: 5'-GCAATGAAATTTCCATTACTCTC-3'). The PCR program was set as: 60°C for 1 min, 42°C for 10 min, 50°C for 30 min, and 94°C for 15 min followed by 35 cycles of 94°C for 30 sec, 50°C for 30 sec, and 72°C for 1 min and lastly followed by 72°C for 10 min. The size of this PCR product was 456 bp and was resolved in 1.2 % agarose gels. PCR products were sequenced directly to confirm the identity of the products.

## Results and Discussion

### Establishment of a new H5N1 PCR primer set

**Figure 1:**
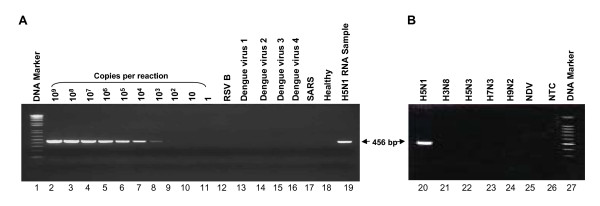
**Detection of H5N1 avian influenza A virus by one-step RT-PCR**. A. Amplification of serially diluted *in *vitro-transcribed single-stranded RNA (lanes 2 to 11) measured by RiboGreen RNA quantitation reagent and H5N1 RNA extracted from allantoic fluid of infected egg (lane 19). The non-template control is (sterile water) illustrated as "NTC". The viral load is indicated by the number of copies per reaction, (lane 2) 1 × 10^9 ^copies per reaction, (lane 3) 1 × 10^8 ^copies per reaction, (lane 4) 1 × 10^7 ^copies per reaction, (lane 5) 1 × 10^6 ^copies per reaction, (lane 6) 1 × 10^5 ^copies per reaction, (lane 7) 1 × 10^4 ^copies per reaction, (lane 8) 1 × 10^3 ^copies per reaction, (lane 9) 1 × 10^2 ^copies per reaction, (lane 10) 10 copies per reaction, and (lane11) 1 copy per reaction. Viral RNA extracted from human specimens that were previously confirmed respiratory syncytial virus (RSV) B (lane 12), dengue virus 1 (lane 13), dengue virus 2 (lane 14), dengue virus 3 (lane 15), dengue virus 4 (lane 16) and severe acute respiratory syndrome (SARS) (lane 17) were also tested as known negatives. RNA extracted from healthy individual (lane 18) was also performed. B. Specific detection of H5N1 avian influenza A virus. Reference strains of different subtypes of avian influenza A viruses (lanes 20 to 24), including non-influenza viruses such as Newcastle disease virus (NDV, lane 25) are indicated. The H5N1, H3N8, H9N2 and NDV isolates were isolated from field samples by the Veterinary Research Institute, Malaysia, the H5N3 and H7N5 isolates were provided by the Department of Veterinary Pathology of Tottori University, Japan. Negative signals from non-H5N1 isolates and the non-template control (water) are shown.

Primers were designed at the conserved regions of the viral HA gene which may be less likely to be affected by mutational changes. This allows the detection of a broad range of isolates and variants of the H5N1 subtype. The performance of the primers was first assessed in gel-based assays using *in vitro*-transcribed RNA generated by the T7 RiboMax Express *in vitro *transcription system (Promega, USA). The concentration of purified transcribed RNA was measured by RiboGreen RNA quantitation reagent (Invitrogen, USA) and serial dilutions of *in vitro*-transcribed RNA were prepared in duplicate. A single one-step RT-PCR was done using 2 μl of RNA in a thermal cycler as described in Methods, and products were analyzed on agarose gels. Non-template controls were included. The sensitivity of the assay was found to be less than 1 × 10^3 ^copies and was able to specifically detect H5N1 RNA (Fig. 1A, lanes 2 to 11).

### Specific detection of H5N1 avian influenza A

**Figure 2:**
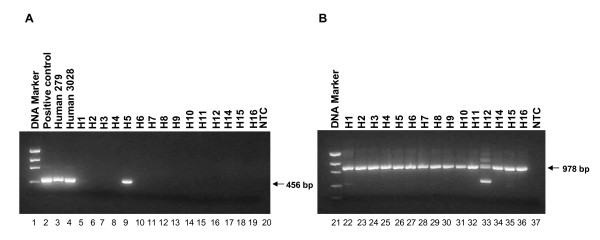
**Specific detection of H5N1 avian influenza A virus from archived human and avian samples**. A. A panel of archived avian and human subtypes (H1 to H16) of Influenza A virus was tested against the H5N1 primers. Viral RNA were extracted from H1 (lane 5, A/Duck/Victoria/23/81); H2 (lane 6, A/Singapore/1/57); H3 (lane 7, A/Sydney/5/97); H4 (lane 8, A/Stint/Australia/1/2004); H5 (lane 9, A/Avian/L2640C); H6 (lane 10, A/Avian/WA/2727/78); H7 (lane 11, A/Chicken/Victoria/85); H8 (lane 12, A/Turkey/Ontario/6118/67); H9 (lane 13, A/Chicken/Malacca/4905/2003); H10 (lane 14, A/Chicken/Germany/n/49); H11 (lane 15, A/Sandpiper/Australia/6/2004); H12 (lane 16, A/Stint/WA/574/84); H14 (lane 17, A/Mallard/Gurjev/244/82); H15 (lane 18, A/Avian/WA/1762/78); H16 (lane 19, A/Gull/Denmark/68110/62). Two human archived RNA samples, Human 279 (lane 3, A/Hong Kong/279/2003) and Human 3028 (lane 4, A/Vietnam/3028/2004) were tested positive. Positive H5N1 RNA sample from *in *vitro-transcribed single-stranded RNA was used (lane 2), and the non-template control (water) is shown. B. The same set of RNA samples were tested against the matrix gene. The 978 bp fragment was detected in all samples (lanes 22 to 36) as shown.

To establish the specificity of the assays for H5N1 subtype detection, we then tested the primers on several known strains of influenza A viruses derived from avian sources (H3N8, H5N3, H7N3 and H9N2). Non-influenza viruses such as Newcastle disease virus (NDV) were also included as controls (Fig. 1B). Thirteen of other known human respiratory diseases caused by viruses and bacteria were also included (Fig. 1A, lanes 12 to 17, and Table 1) as controls. Results showed that detection was specific to H5N1 only (Fig. 1A and 1B). As the number of actual human avian influenza virus infected cases are extremely sparse during this study, the inclusion of a panel of known human samples that do not contain H5N1 virus derived from patients exhibiting "flu"-like symptoms are very helpful in demonstrating the non cross-reactive nature of these primers. In an effort to further investigate the specificity of this set of primers, a panel of avian and human subtypes (H1 to H16) of Influenza A virus was screened. Results showed that there was reactivity only against the H5 subtypes with this primer set and no reactivity against the other subtypes (Fig. 2A, lanes 2 to 19). To ensure that the RNA from these subtypes were not degraded, the matrix gene from these samples were amplified in parallel. As expected, the 978 bp product was amplified for all the subtypes tested (Fig. 2B, lanes 22 to 36). A total of 145 field samples comprising of known and suspect cases from chickens, ducks and muscovies isolated from Vietnam and Malaysia during the 2004 to 2005 outbreak were tested for H5N1 RNA (Table 2). Samples ranged from homogenized pooled organs and tissues, allantoic fluid, cloacal and tracheal swabs. All yielded positive results with 100% positive detection for allantoic fluid, 67% for cloacal and tracheal swab, and 86% for homogenized pooled tissue and organs (Table 2) verifying the sensitivity of this RT-PCR assay. Viral culture isolation methods performed in eggs were used as confirmatory tests for all positive samples (Table 2). This variation in detection could be due to the different efficiencies in viral RNA recovery from the different samples, with allantoic fluid fractions having the highest efficiency for H5N1 RNA recovery and detection among the samples (Table 2). Optimizing the extraction protocols may also improve the RNA recovery from other tissues.

**Table 1:** Human specimens used as controls in one-step RT-PCR H5N1 assay

**Pathogen**	**Early disease symptoms**	***n***
***Virus:***		
Respiratory syncytial virus (RSV) B	"Flu"-like	1
Dengue 1	Fever, "Flu"-like	1
Dengue 2	Fever, "Flu"-like	1
Dengue 3	Fever, "Flu"-like	1
Dengue 4	Fever, "Flu"-like	1
Severe respiratory syndrome virus (SARS)	High fever, dyspnea, malaise	2
Hepatitis B virus (HBV)	"Flu"-like, malaise	6
***Bacteria:***		
Haemophilus influenzae	Fever, "Flu"-like	1
Legionella pneumopnila	"Flu"-like, pneumonia	1
Klebsiella pneumoniae	"Flu"-like, pneumonia	1
Streptococcus pneumoniae	"Flu"-like, pneumonia	1
Mycoplasma pneumoniae	"Flu"-like, malaise	1
Mycobacterium	Fever, malaise, dyspnea	1

**Table 2:** Detection of avian influenza A H5N1 subtype from field specimens.

**Sample type**	**Source**	**Positive by H5N1 viral isolation**	**Positive by RT-PCR H5N1 primers**	***n***
Allantoic Fluid	Vietnam	100%	100%	58
Cloacal and Tracheal swab	Vietnam	100%	67%	43
Homogenized Pooled tissue and organs	Vietnam and Malaysia	95%	86%	44

**Figure 3:**
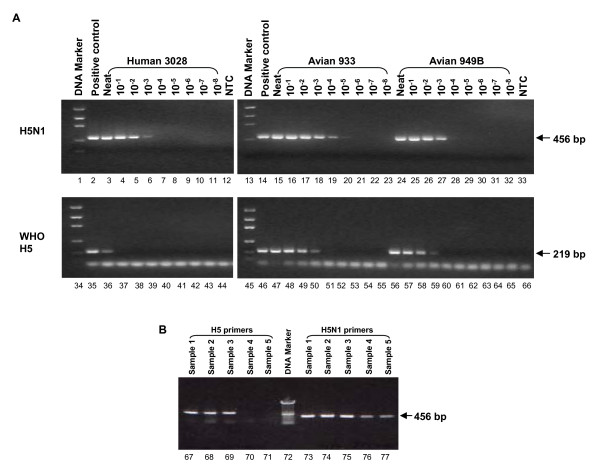
**Sensitivity test against human and avian strains**. A. The H5N1 primers were tested for sensitivity against the currently recommended WHO H5 primer set using three H5N1 strains, one human strain from stored archive RNA, and two freshly extracted avian strains. Human 3028 (A/Vietnam/3028/2004, lanes 3 to 11); Avian 933 (lA/Chicken/Vietnam/933/2004, lanes 15 to 23), and Avian 949B (A/Chicken/Vietnam/949B/2004, lanes 24 to 32) are shown. Serial dilution was performed on the RNA samples from 10^-1 ^to 10^-8 ^as indicated. The 219 bp fragment amplified using the WHO H5 primer set was also performed in parallel as indicated. B. Sensitive detection of H5N1 avian influenza A virus. Five H5N1 samples from Vietnam that have been confirmed by viral isolation and RT-PCR were tested in parallel with an existing H5 primer set (lanes 67 to 71) and the H5N1 primer set (lanes 73 to 77) described in this report. Only three out of five samples were detected by the H5 primer set (lanes 67 to 69), while the new H5N1 primer set detected all five samples (lanes 73 to 77).

In a separate study, the sensitivity of this H5N1 primer set was also evaluated against the currently recommended World Health Organization (WHO) H5 primer set. Three different strains were used – one human archived RNA (Human 3028, from the Vietnam 2004 outbreak) and two freshly extracted avian strains (Avian 933 and Avian 949B). A RNA dilution series was done on the three samples from 10^-1 ^to 10^-8 ^(Fig. 3A, lanes 2 to 11 and lanes 15 to 32), and in all the three strains, the H5N1 primer set described here gave 1 to 2 logs better performance when compared to the recommended WHO primer set (Fig. 3A, Table 3), indicating that this primer set performs well and consistent. In addition, five positive samples from the Vietnam set were also then tested in parallel with another current H5 primer set and results showed that the H5N1 primers described here detected five out of five positive H5N1 samples while the other primer set only managed to detect three out of five (Fig. 3B), indicating that the H5N1 primers described here are more sensitive and could detect weak positive samples with lower viral load.

**Table 3:** Sensitivity of H5N1 primers compared to WHO recommended H5 primers.

**Sample Type**	**Subtype**	**Limit of detection WHO recommended H5 primers**	**Limit of detection H5N1 primers described here**
Human 3028	H5N1	10^-1^	10^-3^
Avian 933	H5N1	10^-1^	10^-3^
Avian 949B	H5N1	10^-3^	10^-4^

Human 3028: A/Vietnam/3028/2004

Avian 933: A/Chicken/Vietnam/933/2004

Avian 949B: A/Chicken/Vietnam/949B/2004

## Conclusion

In conclusion, we have reported an efficient, specific and sensitive assay that has been evaluated on field specimens to be able to detect a wide variety of H5N1 influenza virus isolates. Accurate and sensitive detection of viral RNA is also strongly influenced by the sample type. The rapid one-step single tube reaction described here not only reduce the detection time but also lowers the risk of cross-contamination which has a higher probability in two-steps RT-PCR methods. This cost effective gel-based system has a lower limit of detection in the picogram range which is equivalent to 1 × 10^3 ^copies, and is designed to cater for use in the field in regions where real-time PCR platform and equipments may not be available. Clearly, the results would be further strengthened with the inclusion of more known H5N1 influenza in archived samples from humans, but the availability of human samples are difficult due to the low number of human infections at this point. However, observations from this study strongly suggest that the primers are specific for H5N1 which can be very useful for the early detection and monitoring of avian influenza outbreaks.

## Competing interests

The author(s) declare that they have no competing interests.

## Authors' contributions

LFPN, IB, RSPT, ECR conceived the study, its design and coordination, and results analysis. TN, SMN, LVA, SG, HK, TLT and SSH carried out the experiments and analysis. LFPN and ECR drafted the manuscript.

## Pre-publication history

The pre-publication history for this paper can be accessed here:


